# Alpha desynchronization during simple working memory unmasks pathological aging in cognitively healthy individuals

**DOI:** 10.1371/journal.pone.0208517

**Published:** 2019-01-02

**Authors:** Xianghong Arakaki, Ryan Lee, Kevin S. King, Alfred N. Fonteh, Michael G. Harrington

**Affiliations:** 1 Neurosciences, Huntington Medical Research Institutes, Pasadena, California, United States of America; 2 Imaging Research, Huntington Medical Research Institutes, Pasadena, California, United States of America; Nathan S Kline Institute, UNITED STATES

## Abstract

Our aim is to explore if cognitive challenge combined with objective physiology can reveal abnormal frontal alpha event-related desynchronization (ERD), in early Alzheimer’s disease (AD). We used quantitative electroencephalography (qEEG) to investigate brain activities during N-back working memory (WM) processing at two different load conditions (N = 0 or 2) in an aging cohort. We studied 60–100 year old participants, with normal cognition, and who fits one of two subgroups from cerebrospinal fluid (CSF) proteins: cognitively healthy (CH) with normal amyloid/tau ratio (CH-NAT, n = 10) or pathological amyloid/tau ratio (CH-PAT, n = 14). We recorded behavioral performances, and analyzed alpha power and alpha spectral entropy (SE) at three occasions: during the resting state, and at event-related desynchronization (ERD) [250 ~ 750 ms] during 0-back and 2-back. During 0-back WM testing, the behavioral performance was similar between the two groups, however, qEEG notably differentiated CH-PATs from CH-NATs on the simple, 0-back testing: Alpha ERD decreased from baseline only in the parietal region in CH-NATs, while it decreased in all brain regions in CH-PATs. Alpha SE did not change in CH-NATs, but was increased from baseline in the CH-PATs in frontal and left lateral regions (p<0.01), and was higher in the frontal region (p<0.01) of CH-PATs compared to CH-NATs. The alpha ERD and SE analyses suggest there is frontal lobe dysfunction during WM processing in the CH-PAT stage. Additional power and correlations with behavioral performance were also explored. This study provide pilot information to further evaluate whether this biomarker has clinical significance.

## Introduction

Brain challenge using cognitive tasks have been used to study dynamic brain activities during processing of external information. Analysis of brain activity changes from baseline to the active stage during a cognitive challenge may be helpful to better understand the dynamic abnormalities of information processing in early Alzheimer’s disease (AD) pathology, as has been studied in other neurological conditions, such as schizophrenia, mTBI, migraine [1–3], and symptomatic AD [4–6].

Amyloid pathology is recognized many years before symptomatic or behavioral changes and are early biomarkers of AD [7, 8]. To look at early amyloid pathology in cognitively healthy (CH) individuals, we used cerebrospinal fluid (CSF) measures of amyloid and tau after extensive neuropsychometric testing revealed no cognitive impairment [4]. We thus define elderly **CH** participants with **p**athological CSF **A**beta/**t**au ratio as CH-PAT, and those with normal CSF Abeta/tau ratio as CH-NAT [4]. We performed longitudinal follow-up study of 63 CH elderly participants for 40 months: all 23 CH-NATs remained CH, while 40% of CH-PATs had cognitive decline [9]. Therefore, the CH-PATs are a stage of early AD pathology at greater risk for cognitive decline, and are the focus of this project because they represent a vulnerable population distinct from the CH-NAT that seems to be resilient to cognitive decline.

Working memory (WM) is an executive function among the first neurocognitive domain to be affected in CH-PAT, as in the Stroop Color Word Interference test [4]. WM refers to the cognitive activity to transiently store and manipulate information in real time [10]. WM can be easily assessed by (visual) N-back testing. Brain networks activated during N-back tests have been revealed by brain imaging [11, 12]. However, the mechanisms by which brain resources are allocated and integrated to support WM functions, and the extent these processes are compromised in the CH-PAT stage compared with CH-NAT, are not known. Significant differences in oscillatory activities and event-related potentials (ERPs) during WM testing have been reported between mild cognitive impairment (MCI) and cognitively healthy individuals [13], leading us to challenge cognitive function in CH-PAT to unmask early AD pathophysiology.

Early AD pathology includes synaptic dysfunctions [7, 8, 14–16]. Electroencephalography (EEG) recordings with high temporal resolution can identify cerebral oscillatory dynamic changes and synaptic dysfunctions in the WM network, and so are well-suited to the study of early AD neurodegeneration [7, 8, 16]. Oscillatory activity in the alpha band (8–12 Hz or 8–15 Hz) is the dominant oscillation in healthy, awake humans and responds to a stimulus with both a decrease and increase in power, such that the alpha frequency event-related desynchronization (ERD) is followed by event-related synchronization (ERS) [17]. Alpha ERD is related to memory storage [18, 19], “neural efficiency”, and intelligence [20–22], as well as WM capacity [23]. Alpha ERD during WM is associated with fronto-parietal network activity, and supports the alpha oscillation relationship to top-down network interactions [24, 25], as shown in concurrent EEG and functional magnetic resonance imaging (fMRI) recordings [26]. Similar associations have been found in attention deficit/hyperactivity disorder (ADHD) studies [24, 27]. Therefore, alpha ERD during WM testing was considered an active stage of WM processing [17, 23], and may therefore enlighten our understanding of dynamic brain processing changes in the CH-PAT stage.

In addition to oscillatory activity, EEG complexity has also been studied in neurological conditions. One of the EEG complex parameters, spectral entropy (SE), derived from Shannon entropy (measures probability distribution), allows for quantifying the degree of disorder in EEG power spectral density [28]. As a measure of signal complexity, SE values range from 0 to 1. High SE implies broad spectral content, or a more variable, “irregular”, or “noisy” signal; whereas low SE indicates a narrow frequency range, or a more “regular” signal [29]. Compared to Shannon entropy, SE provides greater resolution in the frequency domain, and allows analysis of spectral entropy contributions from specific frequency ranges [30, 31], such as the alpha band. SE values of EEG frequency bands reflect the coordination of intra- or inter-regional brain activities from neuronal oscillations. When different brain regions are recruited in a challenge task, the participants may have different SE values. Therefore, alpha SE studies may add to our understanding of dynamic brain processing during WM testing in CH-PAT compared with CH-NAT.

Our exploratory study aimed to explore how cerebral alpha power changes in CH-PAT. We analyzed alpha ERD activity in a visual N-back WM paradigm to examine differences in activity changes between CH-NAT and CH-PAT participants. We specifically focused on alpha power and SE in the 8–15 Hz range during resting, baseline, and the ERD time window upon WM challenge. We find that alpha ERD and alpha SE are abnormal at different sensors or brain regions in CH-PATs, and that alpha ERD at sensor cluster over frontal cortex as well as at the most representative sensor Fp2 correlate with CSF classifications. Because frontal hyperactivity has been reported previously in an early AD stage [32–36], we hypothesized that the WM brain challenge would result in lower (more negative) frontal alpha ERD in CH-PATs than in CH-NATs. The purpose of this exploratory pilot study is to provide pilot information for further clinical significance evaluation.

## Materials and methods

### Participants

The Huntington Medical Research Institute (HMRI) Institutional Review Board (IRB) approved this study (Quorum IRB, Seattle, Study # 27197). All participants signed IRB approved consent for this study.

Twenty seven cognitively healthy participants whose ages ranged from 60 to 100 years were recruited for the pilot study. Sources of recruitment were: an internal list of previously classified CH-NATs and CH-PATs (an equal number of each, and unknown to the investigator), advertisements placed in local newspapers and newsletters, the Pasadena Huntington Hospital Senior Health Network, the Pasadena Senior Center, and meetings with local physicians where we presented this research. Three potential participants were excluded because of age or clinical classification (MCI), resulting in 24 study participants: 10 NATs and 14 PATs. EEG Data collection and analysis were performed while blinded to group classification. Assessments included collection of demographic data, physical exam, blood work, disease severity and disability scales, and CSF amyloid/tau measurements [4]. Participants with any cognitive impairment, i.e., global clinical dementia rating scale (CDR) scores > 0.0, were excluded. Only participants who had Uniform Data Set format examination with no classifiable psychiatric or neurological disorder were diagnosed as CH and enrolled in this study after a five-hour comprehensive neuropsychological battery in which testing was performed independent/blind to the biochemical classification. We test the cognitive domains of memory, executive function, language, attention, and visuospatial orientation, and all data was normalized to age, sex, and education normative tables [4]. These formal neuropsychometric data were combined with clinical dementia rating, Montreal Cognitive Assessment, Mini Mental State Examination, as described [4]. Participants were then divided depending on individual CSF Aβ/tau ratios compared to a cutoff value derived from a logistic regression model that correctly diagnosed >85% of clinically probable AD participants [4].

### Procedures

Study participants were seated in a quiet room, and were first asked, for resting state baseline measures, to “sit still” and “empty their minds” for 5 minutes with eyes open (eyes fixed at the DELL sign on the bottom of the dark screen), and then for 5 minutes with eyes closed.

The brain cognitive challenge, or N-back WM tests (N = 0, 2 that reflect the load conditions of the task), was administered using E-prime software (Psychology Software Tools, Inc., Sharpsburg PA) on a Dell Precision T5610 with a 20” screen. Although different types of stimuli can be used for WM, in this study we used letters [37]. Procedures were similar as previously described [38]. Briefly, participants were comfortably seated before a computer screen and were instructed, practiced for 2–3 minutes, and then tested for 0-back, then for 2-back. Each load condition included 3 blocks of 30 trials a block. The N-back task took about 12–25 minutes to complete, depending on each participant’s performance.

### EEG recordings

Online EEG data were collected during resting or during WM challenge as previously described [38]. Briefly, a 21-sensor, dry electrode system (Quasar Wearable Sensing, DSI-24, San Diego, CA) was placed approximately at locations at the international 10–20 system (Fp1, Fp2, F7, F3, Fz, F4, F8, T3, C3, Cz, C4, T4, T5, P3, Pz, P4, T6, O1, O2, M1, and M2) and used to record EEG signals. EEG signals were sampled at 300 Hz, and bandpass filtered between 0.003–150Hz. Three auxiliary sensors were used to record electrooculographic (EOG), electrocardiographic (ECG), and electromyography (EMG) (on the right forearm) activities. A trigger channel encoded the time of presentation of the letter stimuli, the participants’ responses, and the type of test (0- or 2-back) for further analysis.

### Behavioral and EEG data processing

All behavioral and EEG data collection and processing were performed blind to the CH-NAT/CH-PAT status. The behavioral performances were described and compared by accuracy (ACC) and response time (RT): ACC was defined as the percentage of correctly responded trials out of the total trials; RT as the duration from stimulus onset to participant’s response.

All datasets were analyzed in EEGLAB version 13.4.3b [39] running in MATLAB R2016b (The MathWorks, USA) and custom codes developed in-house. The continuous EEG recordings were segmented into epochs of 2500 ms duration during eyes closed for resting state, or using the stimulus onset as a reference during WM, including 500 ms before and 2500 ms after the stimulus onset. Only correctly responded trials were used for analysis because we were interested in activities that are supported by the WM task. Preprocessing and time-frequency (TF) analyses were as previously described [38]. Briefly, epochs were filtered between 2 and 30 Hz, and independent component analysis (ICA) [39] was used to remove eye blinks and cardiac and other muscle artifacts. Large artifact activity greater than three standard deviations (SDs) from the mean of a specific sensor, were rejected. For TF analysis, epoched EEG data were decomposed with logarithmic scaling between 2 and 30 Hz by fast Fourier transform and Morlet wavelet [$e^{i2\pi tf}e^{-t^{2}/{2\sigma }^{2}}$] convolution in the frequency domain, followed by the inverse fast Fourier transform [40, 41]. Power values were normalized by decibels to the baseline power from -400 to -100 ms pre-stimulus at each frequency band [$dB\;power=10*log10(\frac{power}{baseline})$]. Based on the TF plots and published data [42–44], alpha ERD (range 250–750 ms, 8–15 Hz) were then extracted for comparison across sensors, participants, and groups. This was done separately for each sensor, condition, and participant.

Besides alpha ERD, other frequency bands, such as theta and beta bands, are also reported to be important for WM and different load conditions [17, 45]. Therefore, theta (4–8 Hz), alpha (8–15 Hz), and beta (15–30 Hz) bands at the early [0 to 1000] ms window (except alpha at [250 to 750] ms) or late [1000 to 2500] ms window were compared between CH-NAT and CH-PAT participants. The relationship between power and behavioral performance (ACC and RT) were studied using Pearson’s correlation.

### Spectral entropy (SE) analysis

EEG values from the [0 ~ 2000] ms time window were analyzed from resting EEG measurements. Consistent with alpha power quantification, alpha SE for baseline EEG was analyzed using the [-400 to -100] ms time window between active tasks of N-back memory test trials, and alpha SE for active (ERD) EEG was calculated using the [250–750] ms time window. The SE of every EEG channel was calculated at each time point in the respective temporal windows of each EEG condition using the following formula: $SE=\frac{1}{\ln\left(N\right)}\sum_{f_{i}=f_{1}}^{f_{2}}P_{n}(f_{i})\ln\left(\frac{1}{P_{n}(f_{i})}\right)$ [46], where *N* is the number of frequency components in the [f1 f2] range, with f1 and f2 being the lower (8 Hz) and upper (15 Hz) limit of the alpha frequency band respectively, and *P_n_*(*f_i_*) is the normalized power spectrum.

### Statistical methods

Group comparisons on participant baseline characteristics were done using two-sided t-tests or Fisher’s exact test. For each participant, alpha power and alpha SE values from each sensor were averaged for each of the following 5 regions [2, 38]: frontal or F (Fz, F3, F4), central or C (Cz, C3, C4), parietal or P (Pz, P3, P4), left lateral or LL (F7, T3, T5), right lateral or RL (F8, T4, T6). All alpha ERD data are normally distributed. The alpha power was compared using ANCOVA with 3-level TASK (rest, 0, 2-back) and 5-level electrode (F, C, P, LL, RL) as within-subject and two level group (PAT, NAT) as between-subject factors. Further, since frontal hyperactivity has been reported in early AD [32–36], we hypothesized that the WM brain challenge would result in lower (more negative) frontal alpha ERD in CH-PATs than in CH-NATs. We compared frontal alpha power under three conditions (resting state, 0-back, and 2-back) and between two groups (CH-NATs and CH-PATs), using mixed model repeated measures analysis. Alpha power and SE for each region were compared between groups using two-sided t-tests. Analyses were done using PRISM v6.07 (GraphPad) or Excel from Microsoft Office 2013. Since this was an exploratory study, a significance level of 0.05 was used for all tests.

## Results

### Study participant demographics

Ten of 24 participants had normal Aβ/tau ratios (CH-NAT) and 14 had abnormal Aβ/tau ratios (CH-PAT). CH-NATs and CH-PATs were similar on age, gender, education, and handedness (Table 1), suggesting that these factors were not likely contributors in any behavioral and EEG measures described below.

**Table 1:** Mean (SD) baseline characteristics of participants.

		CH-NAT (n = 10)	CH-PAT (n = 14)	p-value
Mean Age (SD)	Mean (SD)	77.8 (8.2)	80.4 (7.2)	0.44^&^
Gender [n (%)]	Female	7 (70%)	8 (57.1%)	0.68^#^
	Male	3 (30%)	6 (42.9%)	
Mean Education (SD) (yrs)		17.1 (2.1)	17.4 (2.3)	0.72^&^
Handedness [n (%)]	R	9 (90%)	11 (78.6%)	0.61^#^
	L	1 (10%)	3 (21.4%)	

Abbreviations: R/L, right/left; SD, standard deviation.

^&^ Two-tailed t-test

^#^ Fisher's exact test.

### Behavioral performance (ACC and RT)

For the 0-back test, neither ACC nor RT were significantly different between the CH-NAT and CH-PAT participants (Table 2). In contrast for the 2-back test, ACC was significantly better in CH-NAT compared to CH-PATs; RT for the 2-back test was not different between the two groups.

**Table 2:** Mean (SD) response accuracy (ACC) and response time (RT) in N-back WM.

	CH-NAT	CH-PAT	P value
0-back			
N	10	14	
ACC	0.90 (0.06)	0.88 (0.06)	0.44
RT (ms)	574.45 (96.40)	559.73 (63.23)	0.68
2-back			
N	10	14	
ACC	0.82 (0.07)	0.75 (0.09)	0.03*
RT (ms)	865.35 (128.02)	836.85 (132.36)	0.6

*P<0.05

### Alpha ERD in midline sensors Fz, Cz, and Pz

**Fig 1:**
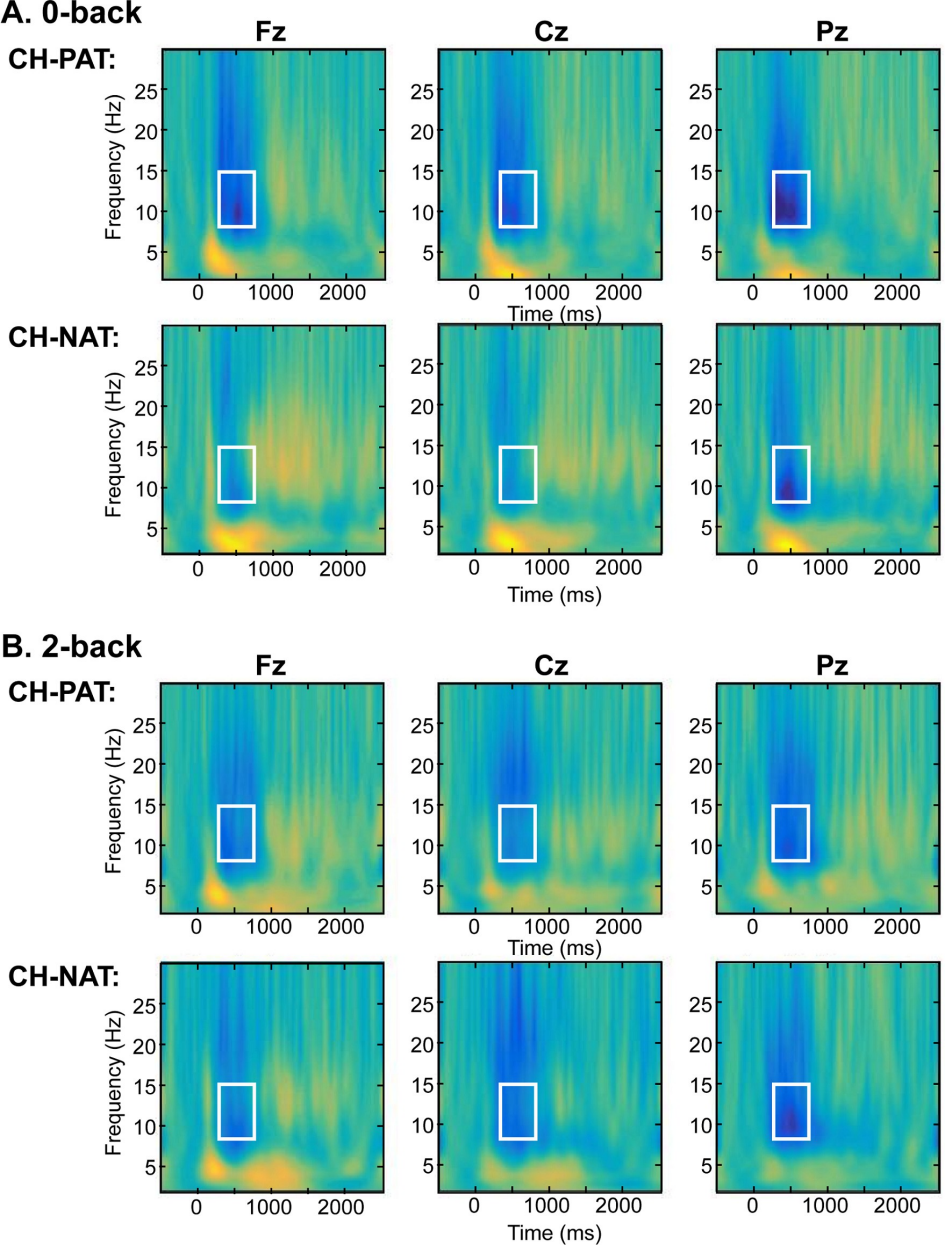
Time-frequency plots (Fz, Cz, and Pz sensors) of mean N-back test. This is a 3D plot with time reference to stimulus onset (x axis in ms), frequency (y axis in Hz), and power (color scale in dB units) during 0-back (A) and 2-back (B) test. The rectangle indicates the representative alpha ERDs (250~750 ms, 8~15 Hz) that were compared between CH-NAT and CH-PAT groups in Fz, Cz, and Pz sensors. Alpha ERDs were lower during 0-back in CH-PAT (-1.93+/-1.77, N = 14) vs. CH-NAT (-0.56+/-1.29, N = 10) participants in Fz sensor, p = 0.0498. Column 1 shows power from Fz, column 2 Cz, and column 3 Pz.

To show a general fronto-posterior distribution of alpha ERD during the 0-back WM test, Fig 1 shows a comparison (CH-NAT vs. CH-PAT) in time frequency plots of mean power of EEG at the saggital plane midline sensors Fz, Cz, and Pz. Power is displayed in the color scale. During 0-back, despite “normal” behavioral performance measures (Table 2), total power of alpha ERD (250~750 ms, 8–15 Hz) in the Fz sensor was significantly lower (p = 0.0498) in the CH-PAT group compared to CH-NATs, as evidenced in the boxed region in Fig 1A (more negative, blue color). There is no significant difference between the two groups during 2-back (Fig 1B). Quantitative results are in Tables 3 & 4.

**Table 3:** Comparison of alpha ERD in the Fig 1 boxed regions between CH-NAT and CH-PAT during 0-back test.

	CH-NAT (n = 10)		CH-PAT (n = 14)		P value
	Mean	SD	Mean	SD	
Fz	-0.56	1.29	-1.93	1.77	**0.0498**
Cz	-0.67	1.18	-1.77	1.35	0.0501
Pz	-1.54	1.40	-2.14	1.40	0.31

**Table 4:** Comparison of alpha ERD between CH-NAT and CH-PAT during 2-back test.

	CH-NAT (n = 10)		CH-PAT (n = 10)		P value
	Mean	SD	Mean	SD	
Fz	-1.25	1.92	-1.50	1.70	0.762
Cz	-1.60	1.80	-1.21	0.89	0.547
Pz	-2.00	1.96	-1.81	1.34	0.804

### Alpha baseline values

**Fig 2:**
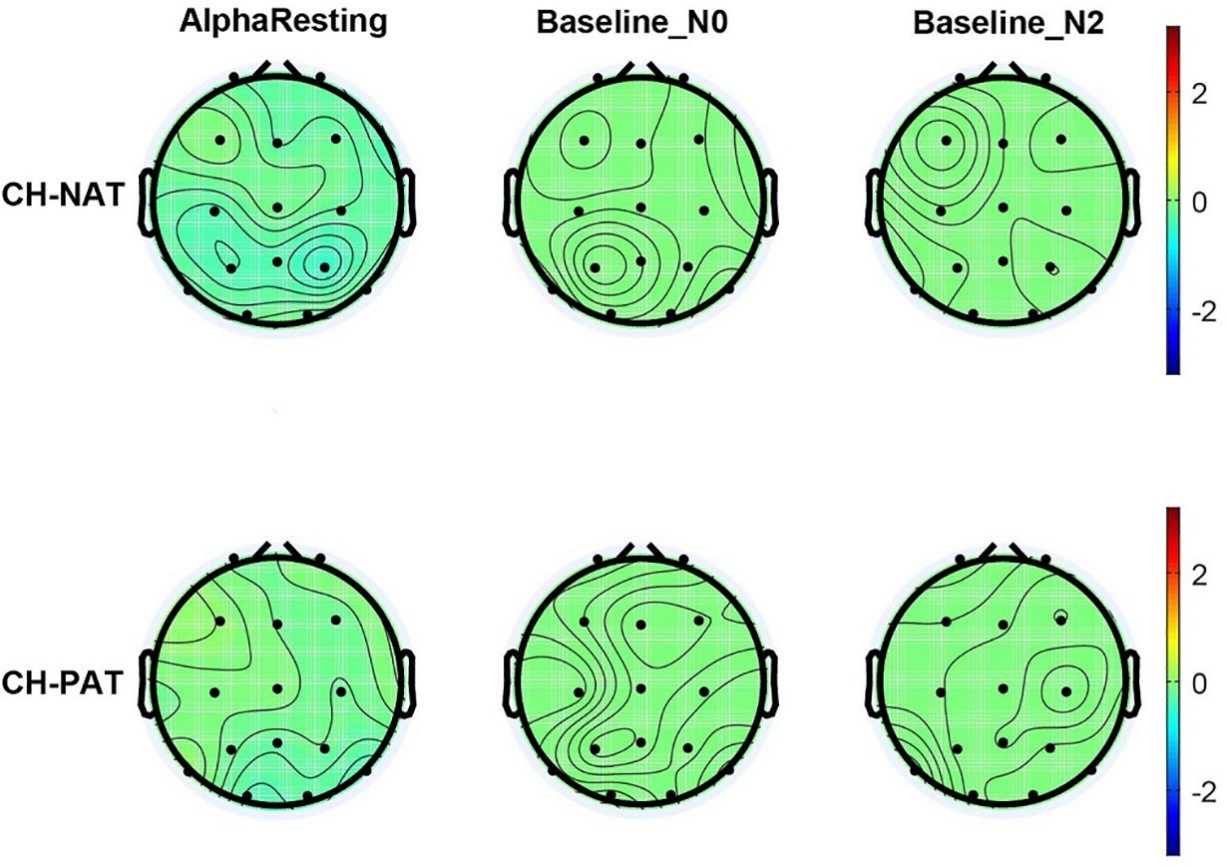
Topoplots of mean alpha power during resting, 0-back baseline, and 2-back baseline. Alpha power in dB units is based on the color scale on the right side. There were no significant differences between CH-NAT and CH-PATs, either during resting, 0-back baseline, or 2-back baseline.

Fig 2 compares CH-NAT and CH-PAT participants on time frequency plots of mean alpha power during resting, as well as during 0- and 2-back baselines. The figure and descriptive data suggest that baseline alpha power was comparable between CH-NATs and CH-PATs, during resting, 0-back baseline, and 2-back baseline (Table 5).

**Table 5:** Comparison of mean (SD) alpha power during resting and WM baseline.

		CH-NAT	CH-PAT	P value
Resting	F	-0.15 (0.52)	-0.07 (0.44)	0.71
	C	-0.23 (0.48)	-0.10 (0.48)	0.54
	P	-0.40 (0.68)	-0.18 (0.48)	0.36
	LL	-0.18 (0.37)	0.01 (0.39)	0.25
	RL	-0.26 (0.39)	-0.13 (0.39)	0.43
Baseline_N0	F	-0.04 (0.02)	-0.05 (0.04)	0.41
	C	-0.04 (0.02)	-0.04 (0.03)	0.92
	P	-0.05 (0.02)	-0.05 (0.03)	0.73
	LL	-0.04 (0.02)	-0.04 (0.02)	0.66
	RL	-0.04 (0.02)	-0.05 (0.02)	0.63
Baseline_N2	F	-0.06 (0.09)	-0.05 (0.03)	0.72
	C	-0.05 (0.06)	-0.05 (0.02)	0.98
	P	-0.04 (0.03)	-0.05 (0.03)	0.58
	LL	-0.04 (0.03)	-0.06 (0.04)	0.16
	RL	-0.04 (0.03)	-0.04 (0.02)	0.97

### Alpha ERD differences

**Fig 3:**
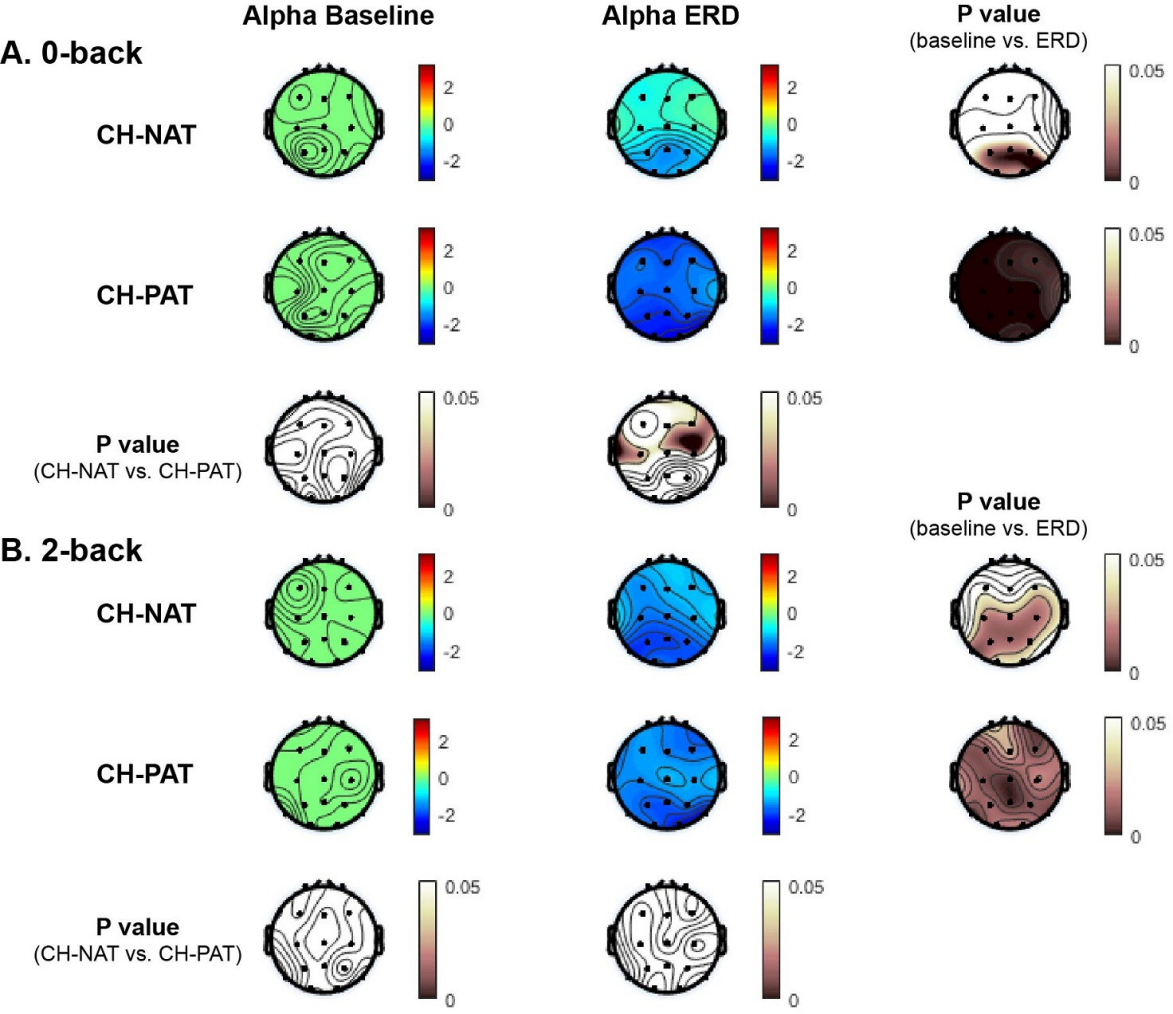
Topoplots of mean alpha power during baseline and during ERD for different load condition, by CSF group. Alpha power is in dB units based on the colored scale bar on the up-left region on each panel; p values based on the pink scale bar are on the right and bottom side A and B. Alpha power during 0-back (A) and 2-back (B) at baseline and ERD window in the two groups CH-NAT and CH-PATs (p values of the within group differences are shown in the right column; p values of the between group differences are shown on the bottom rows in A & B).

Alpha ERD differences during 0-back are shown in Table 6 and Fig 3A. During 0-back, significant decrease in alpha power during ERD time window was confined to the parietal region (p = 0.003) in the CH-NAT group, while alpha power was significantly decreased during ERD window in the CH-PAT group in all 5 regions (frontal, central, parietal, left lateral, and right lateral) (p < 0.001). With regard to our hypothesis, frontal alpha ERD was significantly lower in the CH-PAT than in the CH-NAT group (p = 0.038). Additionally, alpha ERD over the central, left lateral, and right lateral regions was also significantly lower.

**Table 6:** Mean (SD) alpha power at baseline and ERD during 0-back.

		baseline	ERD	P value (baseline vs. ERD)	P value (ERD CH-NAT vs. CH-PAT)
CH-NAT	F	-0.04 (0.02)	-0.57 (1.21)	0.181	**0.038**
	C	-0.04 (0.02)	-0.62 (0.99)	0.080	**0.016**
	P	-0.05 (0.02)	-1.37 (1.21)	**0.003**	0.217
	LL	-0.04 (0.02)	-0.77 (1.15)	0.058	**0.025**
	RL	-0.04 (0.02)	-0.63 (1.29)	0.164	**0.027**
CH-PAT	F	-0.05 (0.04)	-1.76 (1.40)	**0.0001**	
	C	-0.04 (0.03)	-1.76 (1.13)	**<0.0001**	
	P	-0.05 (0.03)	-2.04 (1.36)	**<0.0001**	
	LL	-0.04 (0.02)	-1.97 (1.26)	**<0.0001**	
	RL	-0.05 (0.02)	-1.96 (1.43)	**<0.0001**	

Alpha ERD differences during 2-back are shown in Table 7 and Fig 3B. Alpha power significantly decreased during ERD time window in the CH-NAT group in central and parietal regions (p = 0.008), while alpha power significantly decreased during ERD window in CH-PATs in all 5 regions (p<0.005). However, these differences between CH-NAT and CH-PAT groups were not significant.

**Table 7:** Mean (SD) alpha power at baseline and ERD during 2-back.

		baseline	ERD	P value (baseline vs. ERD)	P value (ERD CH-NAT vs. CH-PAT)
CH-NAT	F	-0.06 (0.09)	-1.34 (1.82)	0.040	0.801
	C	-0.05 (0.06)	-1.53 (1.57)	**0.008**	0.743
	P	-0.04 (0.03)	-1.99 (2.05)	**0.008**	0.767
	LL	-0.04 (0.03)	-1.45 (1.79)	0.023	0.830
	RL	-0.04 (0.03)	-1.40 (1.87)	0.033	0.685
CH-PAT	F	-0.05 (0.03)	-1.52 (1.41)	**0.004**	
	C	-0.05 (0.02)	-1.33 (1.06)	**0.001**	
	P	-0.05 (0.03)	-1.75 (1.42)	**0.001**	
	LL	-0.06 (0.04)	-1.60 (1.29)	**0.001**	
	RL	-0.04 (0.02)	-1.71 (1.47)	**0.002**	

To avoid multiple-comparison bias, alpha power was also compared using ANCOVA. Table 8 shows the least square mean difference, demonstrating the only significant differences were for 0-back, for all regions except the parietal region: CH-PATs had lower alpha power than CH-NATs (estimate = NAT-PAT>0) (p = 0.007~0.021, Table 8). The 3-way interaction (Group*Challenge*Region) was highly non-significant, and the 2-way interactions (Group*Region) and (Challenge*Region) were also not close to being significant. The only significant interaction was Group*Challenge, further demonstrating that CH-NAT and CH-PAT differed by 0-back challenge (p = 0.003, Table 9).

**Table 8:** Statistic summary for alpha power comparison between groups with 3-level challenge (resting, 0-back, and 2-back) and 5 regions (F, C, LT, RT, and P).

Effect	Group	Chall	Region	Group	Chall	Region	Estimate	StdErr	DF	rValue	Probt
Group*Chall*Region	CH-NAT	Resting	C	CH-PAT	Resting	C	-0.13	0.48	110	-0.26	0.796
Group*Chall*Region	CH-NAT	Resting	F	CH-PAT	Resting	F	-0.07	0.48	110	-0.15	0.879
Group*Chall*Region	CH-NAT	Resting	LT	CH-PAT	Resting	LT	-0.19	0.48	110	-0.39	0.700
Group*Chall*Region	CH-NAT	Resting	P	CH-PAT	Resting	P	-0.22	0.48	110	-0.46	0.649
Group*Chall*Region	CH-NAT	Resting	RT	CH-PAT	Resting	RT	-0.13	0.48	110	-0.27	0.790
Group*Chall*Region	CH-NAT	0-back	C	CH-PAT	0-back	C	1.14	0.48	110	2.35	**0.021**
Group*Chall*Region	CH-NAT	0-back	F	CH-PAT	0-back	F	1.19	0.48	110	2.46	**0.016**
Group*Chall*Region	CH-NAT	0-back	LT	CH-PAT	0-back	LT	1.20	0.48	110	2.48	**0.015**
Group*Chall*Region	CH-NAT	0-back	P	CH-PAT	0-back	P	0.67	0.48	110	1.39	0.168
Group*Chall*Region	CH-NAT	0-back	RT	CH-PAT	0-back	RT	1.33	0.48	110	2.75	**0.007**
Group*Chall*Region	CH-NAT	2-back	C	CH-PAT	2-back	C	-0.26	0.51	128	-0.51	0.610
Group*Chall*Region	CH-NAT	2-back	F	CH-PAT	2-back	F	0.13	0.51	128	0.25	0.806
Group*Chall*Region	CH-NAT	2-back	LT	CH-PAT	2-back	LT	0.09	0.51	128	0.18	0.859
Group*Chall*Region	CH-NAT	2-back	P	CH-PAT	2-back	P	-0.30	0.51	128	-0.58	0.560
Group*Chall*Region	CH-NAT	2-back	RT	CH-PAT	2-back	RT	0.25	0.51	128	0.49	0.627

Chall: challenge

**Table 9:** Statistic summary for alpha power comparison between groups with 3-level challenge (resting, 0-back, and 2-back).

Effect	Group	Chall	Group	Chall	Estimate	StdErr	DF	rValue	Probt
Group*Chall	CH-NAT	Resting	CH-PAT	resting	-0.15	0.34	31	-0.43	0.668
Group*Chall	CH-NAT	0-back	CH-PAT	0-back	1.11	0.34	31	3.25	**0.003**
Group*Chall	CH-NAT	2-back	CH-PAT	2-back	-0.02	0.35	34.4	-0.05	0.957

Chall: challenge

Based on frontal hyperactivity in early AD [32–36], we also compared frontal alpha power by groups (CH-NATs and CH-PATs), for the three conditions (resting state, 0-back, and 2-back), using mixed model repeated measures analysis. There are significant different frontal alpha ERD between CH-NATs and CH-PATs during 0-back (p = 0.042, Table 10), but not during resting or 2-back.

**Table 10:** Statistic summary for frontal alpha power comparison between groups with 3-level challenge (resting, 0-back, and 2-back).

	Groups	Chall	Groups	Chall	Estimate	StdErr	DF	tValue	Probt
Group*Chall	CH-NAT	resting	CH-PAT	resting	-0.07	0.20	22	-0.38	0.710
Group*Chall	CH-NAT	0-back	CH-PAT	0-back	1.19	0.55	22	2.16	**0.042**
Group*Chall	CH-NAT	2-back	CH-PAT	2-back	0.04	0.70	21	0.06	0.954

Chall: challenge

### Other frequency band and correlations with behavioral performance

Theta, alpha, and beta power at early and late stages were compared. S1 Table suggest that besides the alpha ERD differences during 0-back shown above, early beta power at the right lateral region during 0-back was lower in CH-PAT than CH-NATs (p = 0.048) (S1 Table), and late theta power at the parietal region during 2-back was higher in CH-PAT than CH-NATs (p = 0.032) (S2 Table). The power correlation with behavioral performance ACC and RT is demonstrated in S3 Table. Briefly, early theta power in CH-PATs but not CH-NATs during both 0-back and 2-back was negatively correlated with 2-back ACC in fronto-parieto-temporal regions (p = 0.001~0.038, r = -0.6~-0.8) (S3 and S4 Tables), while late alpha power in CH-NATs but not CH-PATs during 0-back was positively correlated with 0-back accuracy in fronto-parieto-temporal regions (p = 0.030~0.041, r = 0.6~0.7) (S3 Table).

### Alpha SE baseline values

**Fig 4:**
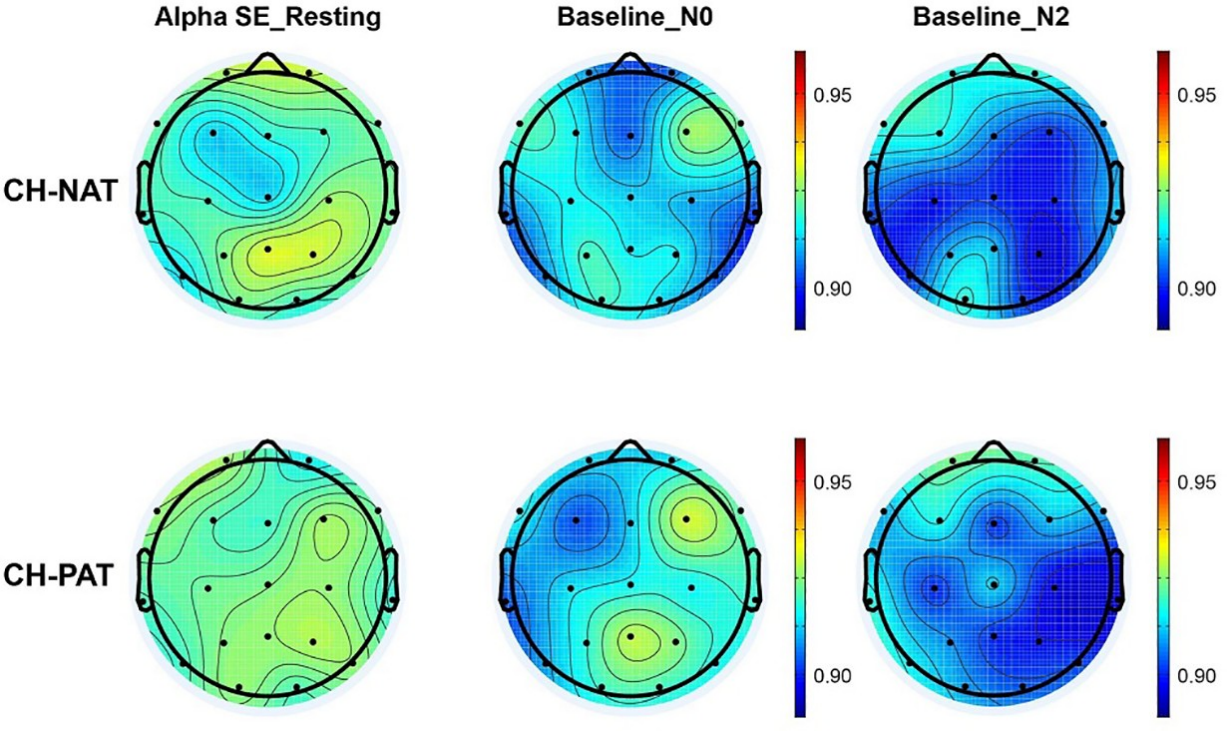
Topoplots of mean alpha spectral entropy (SE) during resting, 0-back baseline, or 2-back baseline. Alpha SE represented by the color scale on the right side. There were no significant differences for alpha SE between CH-NAT and CH-PATs, either during resting, baseline for 0-back, or baseline for 2-back.

Fig 4 compares CH-NAT and CH-PAT participants on mean alpha power spectral entropy (SE) during resting and mean alpha during the 0- and 2-back baselines. The descriptive data suggest that alpha SE are comparable between participants of CH-NAT and CH-PAT groups during resting, 0-back baseline, and 2-back baseline (Table 11).

**Table 11:** Comparison of mean (SD) alpha SE during resting and WM baseline.

		CH-NAT	CH-PAT	P value
Resting	F	0.92 (0.01)	0.92 (0.01)	0.34
	C	0.92 (0.01)	0.92 (0.01)	0.45
	P	0.93 (0.02)	0.93 (0.01)	0.80
	LL	0.92 (0.01)	0.92 (0.01)	0.50
	RL	0.92 (0.01)	0.92 (0.01)	0.88
Baseline_N0	F	0.91 (0.02)	0.92 (0.02)	0.73
	C	0.91 (0.02)	0.92 (0.02)	0.26
	P	0.92 (0.02)	0.93 (0.02)	0.60
	LL	0.91 (0.01)	0.91 (0.02)	0.39
	RL	0.91 (0.01)	0.92 (0.02)	0.39
Baseline_N2	F	0.91 (0.01)	0.91 (0.01)	0.46
	C	0.91 (0.02)	0.91 (0.02)	0.88
	P	0.91 (0.02)	0.91 (0.02)	0.95
	LL	0.91 (0.01)	0.91 (0.01)	0.97
	RL	0.91 (0.03)	0.91 (0.02)	0.92

### Alpha SE differences

**Fig 5:**
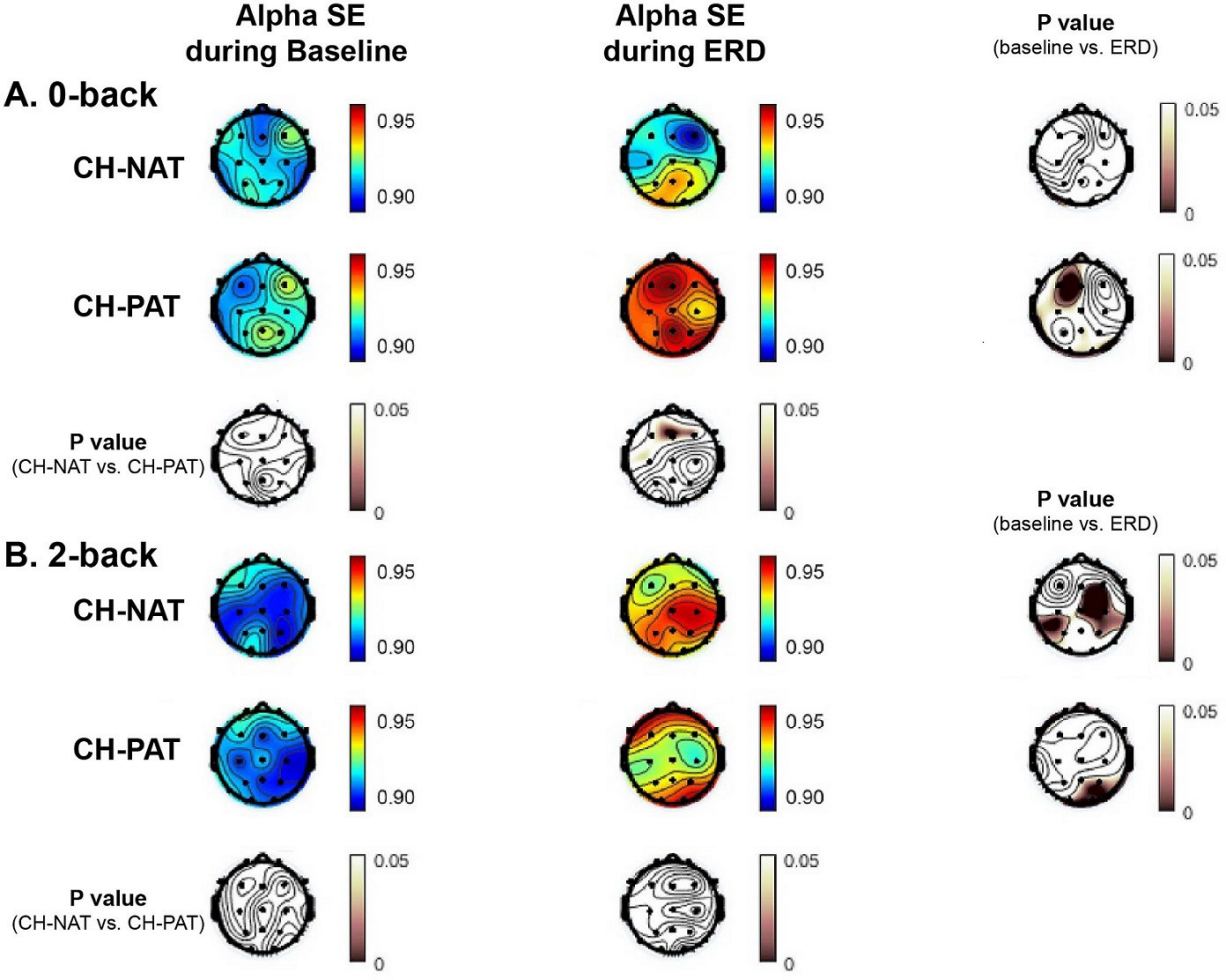
Topoplots of mean alpha SE during baseline and during ERD for different load conditions, by group. Alpha SE was shown in values based on the colored scale bar on up-left region on each panel; p values were shown based on the pink scale bar on the right and bottom side on each panel. Alpha SE during 0-back (A) and 2-back (B) at baseline and ERD window in the two groups CH-NAT and CH-PATs (p values of the within group differences are shown in the right column; p values of the between group differences are shown on the bottom row).

Alpha SE differences during 0-back are shown in Table 12 and Fig 5A. Alpha SE did not significantly change during ERD window in CH-NATs but was significantly increased in CH-PATs in frontal and left lateral regions (p = 0.007 and 0.004, respectively). For the frontal region, this group difference was significant (p = 0.009).

**Table 12:** Mean (SD) alpha SE at baseline and ERD window during 0-back.

		baseline	SE	P value (baseline vs. ERD window)	P value (ERD CH-NAT vs. CH-PAT)
CH-NAT	F	0.91 (0.02)	0.91 (0.03)	0.706	**0.009**
	C	0.91 (0.02)	0.92 (0.03)	0.254	0.233
	P	0.92 (0.02)	0.93 (0.03)	0.314	0.397
	LL	0.91 (0.01)	0.92 (0.02)	0.321	0.105
	RL	0.91 (0.01)	0.93 (0.02)	0.129	0.091
CH-PAT	F	0.92 (0.02)	0.95 (0.03)	**0.007**	
	C	0.92 (0.02)	0.94 (0.04)	0.078	
	P	0.93 (0.02)	0.94 (0.04)	0.124	
	LL	0.91 (0.02)	0.94 (0.03)	**0.004**	
	RL	0.92 (0.03)	0.95 (0.03)	**0.015**	

Alpha SE differences during 2-back are shown in Table 13 and Fig 5B. Alpha SE significantly increased during ERD window in CH-NATs in the central region (p = 0.007) and significantly increased in CH-PATs in the right lateral region (p = 0.003); however, there were no significant group differences.

**Table 13:** Mean (SD) alpha SE at baseline and ERD during 2-back.

		baseline	SE	P value (baseline vs. ERD)	P value (SE CH-NAT vs. CH-PAT)
CH-NAT	F	0.91 (0.01)	0.93 (0.04)	0.174	0.892
	C	0.91 (0.02)	0.94 (0.03)	**0.007**	0.217
	P	0.91 (0.02)	0.94 (0.04)	**0.022**	0.642
	LL	0.91 (0.01)	0.93 (0.03)	**0.037**	0.891
	RL	0.91 (0.03)	0.93 (0.04)	0.113	0.473
CH-PAT	F	0.91 (0.01)	0.93 (0.03)	0.193	
	C	0.91 (0.02)	0.92 (0.03)	0.184	
	P	0.91 (0.02)	0.93 (0.04)	**0.049**	
	LL	0.91 (0.01)	0.94 (0.02)	**0.013**	
	RL	0.91 (0.02)	0.94 (0.03)	**0.003**	

## Discussion

Our study was designed as an exploratory study for potential evidence of brain dysfunction during what is known to be a long, apparently silent period of neurodegeneration before AD symptoms are noticed [4]. Since early cognitive changes are not specific and hard to quantify [4, 47–49], we combined measures of synaptic transmission (qEEG) with a simple WM test to challenge and unmask disrupted brain reserve, analogous with treadmill testing for the heart. The cardiac stress test is a clinically valuable challenge: when an individual at risk for ischemic heart disease with normal resting ECG has a rise in the ST-T segment on ECG during treadmill exercise, it warns of impending coronary occlusion that warrants urgent intervention. In support of our hypothesis, our exploratory data from the brain challenge test has predictive potential: for cognitively healthy older individuals with normal resting EEG, the ERD and SE on qEEG during the simple 0-back WM challenge unmasks abnormal brain activities associated with the CSF amyloid/tau biomarkers of early AD pathology. To further evaluate whether these findings might have clinical significance, we are planning a larger follow-up study to see if our finding of a group difference in frontal ERD is replicable in a new cohort.

During the resting state, the mean values between CH-NATs and CH-PATs differ more than their 0-back or 2-back baseline. The reason is that baseline levels are being normalized to calculate alpha ERD to compare across individuals. The resting states are not completely the same, as they differ based on normal or pathological CSF amyloid/tau. However, this difference is below detectable threshold, p>0.2 for all regions. Therefore, cognitive challenge is necessary to reveal this latent dysfunction in CH-PATs. For the participants, although detailed neuropsychological tests support their CH status, the 0-back alpha ERD and 2-back ACC is significantly different between the two groups (CH-NAT, CH-PAT). Therefore, the results suggest that latent cognitive dysfunction in CH-PATs can be revealed by the WM cognitive challenge, at both electrophysiological (0-back) and behavioral (2-back) level.

Alpha frequency oscillations involve the thalamocortical network, and are essential for information processing in the brain, including attention and WM tasks [17, 26]. For example, alpha ERD relates to encoding and manipulation of spatial representations in WM [50]. Alpha ERD reflects cortical activation, or excitation, and has been correlated with individual WM capacity [17, 23], such that excessive alpha ERD correlates with less WM capacity. Alpha ERD during the WM task was reported to be lower in people with a high intelligence quotient, supporting a higher “neural efficiency” [20–22]. Our alpha ERD values were normalized by decibels to the baseline power, therefore more negative corresponds to more pronounced magnitude and reflects more activation [17, 38, 40, 41]. In our alpha ERD analysis during 0-back, only parietal region was activated in CH-NAT, while in CH-PAT the whole brain activated, indicating lower WM capacities in CH-PAT, which could correlate with a lower “neural efficiency” in participants with abnormal CSF amyloid/tau ratio. During 2-back, both groups showed widespread whole brain activation during ERD, while CH-PAT seemed saturated or over-taxed, as observed in significantly lower 2-back accuracy compared with CH-NAT. 0-back has been considered a control condition without WM demand [37, 51]. Since both load condition accuracy rates (0- and 2-back) associate with WM neural correlates (ventrolateral prefrontal cortex activation) in a similar older population, 0-back has also been used as a baseline load condition compared to the more complex load condition (2-back) for WM studies [52]. The alpha ERD results during 0-back support our hypothesis, and are consistent with CRUNCH theory: 1) more cortical activation for CH-PATs than for CH-NATs because CH-PATs’ less efficient processing made it necessary to recruit more regions at lower load levels [53]; and 2) under-recruitment in CH-PATs compared to CH-NATs during 2-back support 2-back being high set size for CH-PATs. Hyperactivity in alpha band (frontotemporal region) was also reported in a progressive MCI population compared with stable MCI or healthy aging group over the parietotemporal region by magnetoencephalography during a modified Sternberg’s memory task [54]. To avoid multiple comparison bias, our ANCOVA analysis for alpha power demonstrated that the two groups were differentiated by 0-back challenge at all regions except the parietal region.

When we compare the power to ACC and RT, positive correlations were found for late alpha power in CH-NATs during 0-back and 0-back accuracy. This indicates that late alpha reflects performance at low load condition, or less negative alpha ERD (less brain activation) reflects better performance only in CH-NATs. However, no such correlations were seen in CH-PATs. These different correlations suggest that CH-NATs and CH-PATs are using different cognitive strategies to perform the task. We also explored theta correlations with performance, since theta has been shown to relate with frontocortical-hippocampal interplay, is modulated by dopamine [55], and reflects task performance [56]. Negative correlations were found in CH-PATs between early theta during both load conditions (0-back and 2-back) and 2-back accuracy; this indicates that early theta power during the low load can predict performance at higher load conditions, and that early theta power during higher load can reflect performance at the high load condition. That means, higher early theta power during WM can predict or reflect lower performance, supporting a lower neural efficiency in CH-PATs. Again, different correlations for theta power also support that CH-NATs and CH-PATs are using different cognitive strategies to perform the task.

In addition to oscillatory activity, we compared the complexity of EEG signal between CH-PAT and CH-NAT using SE. Shannon entropy serves to quantify the degree of disorder within a system [57], however with limitations in comparing EEG analysis between individuals as a result of inter-individual variability from Shannon entropy not being normalized to the total power of EEG [58, 59]. The idea of SE was developed by Inouye et al. (1991) who called for the application of Shannon entropy to the normalized power spectral density of Fourier-transformed EEG signals [28]. This approach has been implemented to quantify the probability distribution in power field energy across a range of frequencies of the EEG signals, and was used to analyze alpha frequency range in this study.

In our SE analysis during 0-back, the CH-PAT group had significantly higher alpha SE during the active ERD time window than CH-NAT, specifically in the frontal region (p < 0.05), indicating they have more “noisy” brain alpha signals while performing this simple task; this suggests a frontal hyperactivity or compensatory mechanism was detectable in the cognitively healthy group with CSF biochemistry that is consistent with AD-like pathology. This is in line with our alpha ERD power measures. We suggest that the CH-PAT group needed extra brain resources (frontal regions), rather than primarily parietal regions, to filter irrelevant information and process WM because of loss in functional resolution or more disorganization. Simple visual input stimuli to CH-PATs activate greater portions of the brain for recalling and updating mental representations and overall WM processing, further supporting that CH-PATs have lower “neural efficiency”. Thus, both excessive alpha ERD and higher SE indicate the CH-PATs have less WM capacity, or lower neural efficiency.

As the recorded EEG waveforms measure a composite of external electrical activity and synaptic signals of cortical pyramidal neurons, it has been proposed that SE is not just a statistical measure—describing the scope/degree of variability or (ir)regularity and disorder—of EEG signal patterns, but accurately reflects cortical function and “intracortical information flow” [29]. Biologically, it may be more apt to associate entropy with the number of accessible cortical microstates available to the brain rather than a measure of how disordered brain processes are [29]. As such, the greater number of frequency ‘bins’ a signal point depicting brain energy is more likely to fall into in the distribution—as denoted by a higher SE value—can be representative of the greater number of mental microstates accessed by the brain for that particular time point or window.

In contrast to the CH-PAT group, it seems that the task was of such baseline load condition and difficulty that CH-NAT participants were able to manage and perform the 0-back test without the necessity of recruiting additional brain resources or accessing of more mental “microstates.” That corroborates our previous interpretation that CH-PAT participants experience a degree of loss in neural efficiency or functionality. In 2-back test, SE was higher in CH-NATs but not CH-PATs with different behavioral performance, further supporting that more complex load condition 2-back demands greater brain efforts in CH-NATs, while it overtaxes brains of CH-PATs (lower ACC). When CH-NAT and CH-PAT groups showed different frontal SE during 0-back, the SE value ranges are within mean ± 1 SD of each other in all other comparisons in Tables 11–13, supporting all other brain activities are intact to maintain their CH status,

SE has helped quantify the measure of EEG disorder and thereby indirectly describes systemic complexity in terms of the number of accessible mental microstates in the study of several other unique structural and functional changes to the brain—whether as the result of natural decline/deterioration with age [46] or psychiatric, neurological, or less distinctive neuropsychiatric disorders such as epilepsy [31], Alzheimer’s [60], or schizophrenia [61]. However, our study is among the first, to our knowledge, to apply the SE analysis specifically to the alpha frequency band (8–15 Hz) in CH-PAT research.

The frontal abnormalities in this pre-symptomatic stage of early AD pathology are consistent with previous studies in those at a slightly later stage of mild cognitive impairment (MCI), such as the frontal-parietal EEG coupling that differs in patients with MCI from healthy elderlies [62], and the executive decline that was reported to correlate with DTI measurements in the MCI stage [4, 48]. Abnormal frontal functions have been shown in pre-dementia stages of AD. For example, increased functional connectivity in frontal areas was found in progressive MCI or CH-PAT stage [32–35]. This abnormal frontal hyperactivity has been thought to result from compensatory mechanism, or to be caused by inhibitory synaptic decrease from amyloid deposition [63]. Although the p-value is < 0.05 for the Fz sensor results during 0-back, the Fz alpha ERD has significant overlap of the mean ± SD between the CH-NAT and CH-PAT, most likely from a limited number of participants. Therefore, a second larger population could be studied to confirm our findings. Further, our hypothesis testing analysis demonstrated significant different frontal alpha power between CH-NATs and CH-PATs during 0-back, but not during 2-back or resting state. Therefore, 0-back challenge helps bring out the frontal abnormality above detectable threshold from latent dysfunctional resting state in CH-PATs.

Our findings are in line with a recent study, which demonstrated that amyloid deposition related to alpha power increase in the same or adjacent prefrontal areas in CH group [36]. However, our alpha ERD power did not show correlation with CSF amyloid/tau measurements. The reason might be different lab conditions, working procedures, or a limited sample size in our study. Our study supports our hypothesis that predicts frontal abnormalities in this pre-symptomatic AD stage, and provides evidence that this frontal abnormality can be revealed by a simple brain challenge.

Cognitive decline started from preclinical stage, and progressed to MCI and AD [49]. Therefore, detection of early cognitive decline and study of underlying early AD pathology before symptomatic manifestation is critical. Early AD pathology includes early synaptic dysfunction, low Aβ/tau ratio in CSF, and subtle cognitive decline [7]. Synaptic dysfunction, possibly mediated by inappropriate activation of complement-dependent and microglial damage [47], may occur earlier than detectable Aβ deposition [7, 8, 16]. Synaptic dysfunction is not immediately apparent because of various compensatory mechanisms in which additional or different resources are recruited to aid cognitive performance. These compensatory mechanisms are hard to detect by imaging techniques like fMRI (because of temporal resolution limits), and may happen long before cognitive or behavioral symptoms appear. Neurophysiology testing [47], particularly *in vivo* EEG, is used to study synaptic function [64] with high temporal resolution, for example alpha ERD. EEG can be used to understand normal and pathological neural processes responsible for neural and cognitive function, and to monitor response to treatment [65–68]. Compared to resting EEG, the challenge EEG in our study has potential to detect CH-PAT (and inform prognosis) at a time when interventions may have a chance of slowing disease progression.

In AD research, however, EEG studies until now have been limited to symptomatic participants with MCI [69, 70] or only used with behavioral measures in CH-PAT [4]. For example, induced theta activity during N-back is significantly reduced in MCI participants who deteriorate at 1-year follow up, indicating directed-attentional deficits and possible compensatory mechanisms in MCI [69]. Therefore, this study can be significant in that it will help start a way to study AD in this earliest stage using an objective measure.

Our study, combining WM N-back testing with EEG, shows alpha ERD and SE differ between CH-PAT and CH-NAT. Therefore, these results suggest qEEG during simple WM test can help reveal abnormal executive functions in CH-PAT through frontal alpha ERD, and its derived SE. The neuroplasticity that occurs in this CH-PAT stage helps compensate for reduction in neural efficiency for normal task performance, which is difficult to identify during rest or in real life, and can be brought out by the cognitive challenge. Based on our results, we propose that interventions that improve cognitive efficiency at the CH-PAT phase of AD may be helpful to attenuate neurodegeneration. Since gamma frequency has been associated with Alzheimer’s disease [71, 72] and is beyond our frequency range here, we are studying gamma band in a separate analysis.

There are limitations to this study. First, the study was exploratory and results need to be replicated in a bigger population and with longitudinal follow up. Second, it has been demonstrated that preprocessing may distort EEG signals [73]. For example, reference selection can inevitably affect EEG data [74]. We attempted to minimize distortion by using only widely validated pre-processing procedures [39]. Although beyond the scope of the current analysis, future studies using reference electrode standardization technique should be explored [75, 76]. Our SE calculation utilizes the formula to calculate each point in a time window. Although this allows for a degree of temporal resolution while retaining high frequency resolution, it is not exactly time-frequency balanced [29]. Further research should be done on determining the optimal wave cycle number to create the variable sliding time windows for each frequency of the SE analysis to corroborate our findings. Finally, more CH participants are female than male, both for CH-NATs and CH-PATs, which may reflect greater altruism or greater resilience in females for aging. Despite these limitations, our findings regarding alpha ERD and alpha SE during WM task performance provide new insight to early AD pathology and should encourage further related research.

## Conclusions

In this exploratory cross-sectional study, a simple WM paradigm combined with qEEG revealed that frontal neural functionality is compromised in CH-PAT. The results support our hypothesis and suggest that alpha ERD and corresponding SE agree with measurements of established CSF biomarkers of AD. We demonstrate for the first time that neuroplasticity in the CH-PAT stage compensates for reduction in neural efficiency, as revealed by qEEG during cognitive challenge. Moreover, WM testing combined with non-invasive qEEG can be a valuable component of the armamentarium for differentiating early dementia from normal aging, and a useful tool for discovering new therapies.

## Supporting information

S1 Table Early and Late power comparison between CH-NATs and CH-PATs during 0-back. (DOCX)

S2 Table Early and Late power comparison between CH-NATs and CH-PATs during 2-back. (DOCX)

S3 Table Pearson's correlation between early and late power and behavioral (ACC and RT) during 0-back. (DOCX)

S4 Table Pearson's correlation between early and late power and behavioral (ACC and RT) during 2-back. (DOCX)
