# Supplementary material for: Alpha desynchronization during simple working memory unmasks pathological aging in cognitively healthy individuals
Source: PLoS One. 2019 Jan 2;14(1):e0208517. doi: 10.1371/journal.pone.0208517 (PMC6314588; doi:10.1371/journal.pone.0208517)
Supplement: S1 Table — (DOCX) [file pone.0208517.s001.docx]

| **S1 Table. Early and Late power comparison between CH-NATs and CH-PATs during 0-back.** | | | | | | | | | | | | | |
| --- | --- | --- | --- | --- | --- | --- | --- | --- | --- | --- | --- | --- | --- |
|  |  | **early** | | | | |  |  | **Late** | | | | |
|  |  | **CH-NAT** | | **CH-PAT** | |  |  |  | **CH-NAT** | | **CH-PAT** | |  |
|  |  | Mean | SD | Mean | SD | p value |  |  | Mean | SD | Mean | SD | p value |
| **Theta_N0** | F | 0.85 | 0.92 | 0.41 | 1.03 | 0.277 | **Theta_N0** | F | 0.31 | 0.90 | 0.23 | 0.68 | 0.816 |
|  | C | 0.72 | 0.72 | 0.36 | 0.91 | 0.298 |  | C | 0.28 | 0.82 | 0.20 | 0.68 | 0.822 |
|  | P | 0.45 | 0.93 | 0.39 | 0.87 | 0.864 |  | P | 0.19 | 0.69 | 0.14 | 0.68 | 0.861 |
|  | LT | 0.57 | 0.69 | 0.36 | 0.92 | 0.523 |  | LT | 0.41 | 0.68 | 0.15 | 0.54 | 0.335 |
|  | RT | 0.44 | 0.66 | 0.34 | 0.91 | 0.763 |  | RT | 0.04 | 0.68 | 0.12 | 0.58 | 0.762 |
|  |  | **CH-NAT** | | **CH-PAT** | |  |  |  | **CH-NAT** | | **CH-PAT** | |  |
|  |  | Mean | SD | Mean | SD | p value |  |  | Mean | SD | Mean | SD | p value |
| **Alpha_N0** | F | -0.57 | 1.21 | -1.76 | 1.40 | **0.038** | **Alpha_N0** | F | 0.77 | 1.02 | 0.34 | 0.53 | 0.243 |
|  | C | -0.62 | 0.99 | -1.76 | 1.13 | **0.016** |  | C | 0.64 | 0.79 | 0.28 | 0.58 | 0.234 |
|  | P | -1.37 | 1.21 | -2.04 | 1.36 | 0.217 |  | P | 0.41 | 0.70 | 0.22 | 0.67 | 0.516 |
|  | LT | -0.77 | 1.15 | -1.97 | 1.26 | **0.025** |  | LT | 0.71 | 0.86 | 0.33 | 0.60 | 0.239 |
|  | RT | -0.63 | 1.29 | -1.96 | 1.43 | **0.027** |  | RT | 0.50 | 0.77 | 0.42 | 0.53 | 0.775 |
|  |  | **CH-NAT** | | **CH-PAT** | |  |  |  | **CH-NAT** | | **CH-PAT** | |  |
|  |  | Mean | SD | Mean | SD | p value |  |  | Mean | SD | Mean | SD | p value |
| **Beta_N0** | F | -0.19 | 0.49 | -0.60 | 0.54 | 0.068 | **Beta_N0** | F | 0.42 | 0.44 | 0.20 | 0.29 | 0.180 |
|  | C | -0.37 | 0.61 | -0.74 | 0.48 | 0.137 |  | C | 0.32 | 0.38 | 0.23 | 0.34 | 0.581 |
|  | P | -0.31 | 0.64 | -0.72 | 0.53 | 0.117 |  | P | 0.47 | 0.48 | 0.30 | 0.40 | 0.371 |
|  | LT | -0.27 | 0.40 | -0.61 | 0.47 | 0.072 |  | LT | 0.30 | 0.47 | 0.24 | 0.23 | 0.735 |
|  | RT | -0.11 | 0.46 | -0.54 | 0.53 | **0.048** |  | RT | 0.24 | 0.30 | 0.25 | 0.35 | 0.966 |
