# Supplementary material for: Alpha desynchronization during simple working memory unmasks pathological aging in cognitively healthy individuals
Source: PLoS One. 2019 Jan 2;14(1):e0208517. doi: 10.1371/journal.pone.0208517 (PMC6314588; doi:10.1371/journal.pone.0208517)
Supplement: S2 Table — (DOCX) [file pone.0208517.s002.docx]

| **S2 Table. Early and Late power comparison between CH-NATs and CH-PATs during 2-back.** | | | | | | | | | | | | | |
| --- | --- | --- | --- | --- | --- | --- | --- | --- | --- | --- | --- | --- | --- |
|  |  | **early** | | | | |  |  | **Late** | | | | |
|  |  | **CH-NAT** | | **CH-PAT** | |  |  |  | **CH-NAT** | | **CH-PAT** | |  |
|  |  | Mean | SD | Mean | SD | p value |  |  | Mean | SD | Mean | SD | p value |
| **Theta_N2** | F | 0.54 | 1.20 | 0.28 | 0.53 | 0.529 | **Theta_N2** | F | 0.28 | 0.89 | 0.49 | 0.70 | 0.569 |
|  | C | 0.12 | 0.84 | 0.20 | 0.48 | 0.802 |  | C | -0.10 | 0.66 | 0.33 | 0.62 | 0.151 |
|  | P | 0.00 | 0.90 | 0.26 | 0.63 | 0.464 |  | P | -0.05 | 0.53 | 0.47 | 0.45 | **0.032** |
|  | LL | 0.35 | 1.12 | 0.28 | 0.67 | 0.873 |  | LL | 0.09 | 0.70 | 0.45 | 0.55 | 0.219 |
|  | RL | 0.23 | 1.03 | 0.28 | 0.66 | 0.907 |  | RL | 0.02 | 0.70 | 0.36 | 0.52 | 0.231 |
|  |  | **CH-NAT** | | **CH-PAT** | |  |  |  | **CH-NAT** | | **CH-PAT** | |  |
|  |  | Mean | SD | Mean | SD | p value |  |  | Mean | SD | Mean | SD | p value |
| **Alpha_N2** | F | -1.34 | 1.82 | -1.52 | 1.41 | 0.801 | **Alpha_N2** | F | 0.21 | 1.18 | 0.58 | 0.83 | 0.424 |
|  | C | -1.53 | 1.57 | -1.33 | 1.06 | 0.743 |  | C | -0.14 | 0.86 | 0.44 | 0.63 | 0.102 |
|  | P | -1.99 | 2.05 | -1.75 | 1.42 | 0.768 |  | P | -0.27 | 0.78 | 0.45 | 0.76 | 0.052 |
|  | LL | -1.45 | 1.79 | -1.60 | 1.29 | 0.830 |  | LL | 0.21 | 0.87 | 0.45 | 0.70 | 0.509 |
|  | RL | -1.40 | 1.87 | -1.71 | 1.47 | 0.685 |  | RL | 0.08 | 0.79 | 0.55 | 0.68 | 0.170 |
|  |  | **CH-NAT** | | **CH-PAT** | |  |  |  | **CH-NAT** | | **CH-PAT** | |  |
|  |  | Mean | SD | Mean | SD | p value |  |  | Mean | SD | Mean | SD | p value |
| **Beta_N2** | F | -0.79 | 1.49 | -0.77 | 0.57 | 0.973 | **Beta_N2** | F | -0.10 | 1.45 | 0.09 | 0.44 | 0.702 |
|  | C | -1.15 | 1.51 | -0.92 | 0.63 | 0.667 |  | C | -0.34 | 1.41 | 0.02 | 0.41 | 0.457 |
|  | P | -0.84 | 0.81 | -0.80 | 0.62 | 0.896 |  | P | 0.00 | 0.49 | 0.14 | 0.43 | 0.512 |
|  | LL | -0.56 | 0.93 | -0.64 | 0.61 | 0.815 |  | LL | 0.13 | 0.77 | 0.05 | 0.42 | 0.792 |
|  | RL | -0.57 | 0.93 | -0.67 | 0.48 | 0.751 |  | RL | 0.00 | 0.81 | 0.16 | 0.38 | 0.569 |
