# Supplementary material for: Alpha desynchronization during simple working memory unmasks pathological aging in cognitively healthy individuals
Source: PLoS One. 2019 Jan 2;14(1):e0208517. doi: 10.1371/journal.pone.0208517 (PMC6314588; doi:10.1371/journal.pone.0208517)
Supplement: S3 Table — (DOCX) [file pone.0208517.s003.docx]

| **S3 Table. Pearson's correlation between early (_E_) and late (_L_) power and behavioral (ACC and RT) during 0-back.** | | | | | | | | | | | | | | | | | | | | | | | | |
| --- | --- | --- | --- | --- | --- | --- | --- | --- | --- | --- | --- | --- | --- | --- | --- | --- | --- | --- | --- | --- | --- | --- | --- | --- |
|  |  | p | | | | r | | | |  |  | |  | | p | | | | | r | | | | |
| **CH-NAT** |  | ACC0 | RT0 | ACC2 | RT2 | ACC0 | RT0 | ACC2 | RT2 | **CH-PAT** | |  | | ACC0 | | RT0 | ACC2 | RT2 | ACC0 | | RT0 | ACC2 | RT2 |  |
| **Theta_E_N0** | F | 0.428 | 0.597 | 0.399 | 0.339 | 0.3 | -0.2 | -0.3 | -0.3 | **Theta_E_N0** | | F | | 0.890 | | 0.954 | **0.011** | 0.429 | 0.0 | | 0.0 | -0.7 | 0.2 |  |
|  | C | 0.711 | 0.826 | 0.329 | 0.530 | 0.1 | -0.1 | -0.3 | -0.2 |  |  | C | | 0.336 | | 0.444 | **0.014** | 0.969 | -0.3 | | 0.2 | -0.6 | 0.0 |  |
|  | P | 0.336 | 0.960 | 0.814 | 0.325 | 0.3 | 0.0 | 0.1 | -0.3 |  |  | P | | 0.053 | | 0.876 | **0.006** | 0.703 | -0.5 | | 0.0 | -0.7 | -0.1 |  |
|  | LL | 0.640 | 0.395 | 0.265 | 0.106 | 0.2 | -0.3 | -0.4 | -0.5 |  |  | LL | | 0.106 | | 0.747 | **0.001** | 0.551 | -0.5 | | -0.1 | -0.8 | -0.2 |  |
|  | RL | 0.412 | 0.772 | **0.028** | 0.513 | -0.3 | -0.1 | -0.7 | -0.2 |  |  | RL | | 0.253 | | 0.741 | **0.007** | 0.545 | -0.3 | | -0.1 | -0.7 | -0.2 |  |
|  |  | ACC0 | RT0 | ACC2 | RT2 | ACC0 | RT0 | ACC2 | RT2 |  | |  | | ACC0 | | RT0 | ACC2 | RT2 | ACC0 | | RT0 | ACC2 | RT2 |  |
| **Theta_L_N0** | F | 0.763 | 0.776 | 0.174 | 0.555 | 0.1 | -0.1 | -0.5 | -0.2 | **Theta_L_N0** | | F | | 0.202 | | 0.200 | 0.158 | 0.621 | 0.4 | | 0.4 | -0.4 | 0.1 |  |
|  | C | 0.653 | 0.886 | 0.125 | 0.726 | -0.2 | 0.1 | -0.5 | -0.1 |  |  | C | | 0.989 | | 0.091 | 0.193 | 0.523 | 0.0 | | 0.5 | -0.4 | -0.2 |  |
|  | P | 0.668 | 0.867 | 0.621 | 0.524 | 0.2 | -0.1 | -0.2 | -0.2 |  |  | P | | 0.754 | | 0.124 | 0.076 | 0.927 | -0.1 | | 0.4 | -0.5 | 0.0 |  |
|  | LL | 0.334 | 0.274 | 0.588 | 0.494 | 0.3 | -0.4 | -0.2 | -0.2 |  |  | LL | | 0.969 | | 0.150 | **0.037** | 0.690 | 0.0 | | 0.4 | -0.6 | -0.1 |  |
|  | RL | 0.285 | 0.834 | **0.007** | 0.794 | -0.4 | -0.1 | -0.8 | -0.1 |  |  | RL | | 0.723 | | 0.232 | 0.114 | 0.888 | 0.1 | | 0.3 | -0.4 | 0.0 |  |
|  |  |  |  |  |  |  |  |  |  |  | |  | |  | |  |  |  |  | |  |  |  |  |
| **CH-NAT** |  | ACC0 | RT0 | ACC2 | RT2 | ACC0 | RT0 | ACC2 | RT2 | **CH-PAT** | |  | | ACC0 | | RT0 | ACC2 | RT2 | ACC0 | | RT0 | ACC2 | RT2 |  |
| **Alpha_E_N0** | F | 0.615 | 0.277 | 0.411 | 0.644 | 0.2 | -0.4 | -0.3 | -0.2 | **Alpha_E_N0** | | F | | 0.164 | | 0.853 | 0.853 | 0.100 | 0.4 | | 0.1 | 0.1 | 0.5 |  |
|  | C | 0.933 | 0.724 | 0.435 | 0.457 | 0.0 | -0.1 | -0.3 | 0.3 |  |  | C | | 0.302 | | 0.449 | 0.904 | 0.225 | 0.3 | | 0.2 | 0.0 | 0.3 |  |
|  | P | 0.979 | 0.804 | 0.468 | 0.765 | 0.0 | 0.1 | -0.3 | 0.1 |  |  | P | | 0.617 | | 0.380 | 0.995 | 0.288 | 0.1 | | 0.3 | 0.0 | 0.3 |  |
|  | LL | 0.920 | 0.322 | 0.236 | 0.569 | 0.0 | -0.3 | -0.4 | -0.2 |  |  | LL | | 0.728 | | 0.920 | 0.889 | 0.552 | 0.1 | | 0.0 | 0.0 | 0.2 |  |
|  | RL | 0.829 | 0.236 | 0.363 | 0.822 | 0.1 | -0.4 | -0.3 | -0.1 |  |  | RL | | 0.439 | | 0.905 | 0.905 | 0.332 | 0.2 | | 0.0 | 0.0 | 0.3 |  |
|  |  | ACC0 | RT0 | ACC2 | RT2 | ACC0 | RT0 | ACC2 | RT2 |  | |  | | ACC0 | | RT0 | ACC2 | RT2 | ACC0 | | RT0 | ACC2 | RT2 |  |
| **Alpha_L_N0** | F | **0.041** | 0.272 | 0.414 | 0.383 | 0.7 | -0.4 | 0.3 | -0.3 | **Alpha_L_N0** | | F | | 0.294 | | **0.049** | 0.433 | 0.465 | 0.3 | | 0.5 | -0.2 | 0.2 |  |
|  | C | 0.067 | 0.235 | 0.373 | 0.396 | 0.6 | -0.4 | 0.3 | -0.3 |  |  | C | | 0.510 | | 0.614 | 0.260 | 0.559 | -0.2 | | 0.1 | -0.3 | -0.2 |  |
|  | P | **0.037** | 0.249 | 0.223 | 0.514 | 0.7 | -0.4 | 0.4 | -0.2 |  |  | P | | 0.262 | | 0.661 | 0.120 | 0.293 | -0.3 | | 0.1 | -0.4 | -0.3 |  |
|  | LL | **0.030** | 0.121 | 0.369 | 0.361 | 0.7 | -0.5 | 0.3 | -0.3 |  |  | LL | | 0.256 | | 0.683 | 0.118 | 0.259 | -0.3 | | 0.1 | -0.4 | -0.3 |  |
|  | RL | 0.133 | 0.076 | 0.685 | 0.255 | 0.5 | -0.6 | 0.1 | -0.4 |  |  | RL | | 0.381 | | 0.141 | 0.567 | 0.342 | -0.3 | | 0.4 | -0.2 | -0.3 |  |
|  |  |  |  |  |  |  |  |  |  |  | |  | |  | |  |  |  |  | |  |  |  |  |
| **CH-NAT** |  | ACC0 | RT0 | ACC2 | RT2 | ACC0 | RT0 | ACC2 | RT2 | **CH-PAT** | |  | | ACC0 | | RT0 | ACC2 | RT2 | ACC0 | | RT0 | ACC2 | RT2 |  |
| **Beta_E_N0** | F | 0.763 | 0.462 | 0.331 | 0.119 | -0.1 | -0.3 | -0.3 | 0.5 | **Beta_E_N0** | | F | | 0.052 | | 0.842 | 0.266 | 0.576 | 0.5 | | 0.1 | 0.3 | 0.2 |  |
|  | C | 0.498 | 0.798 | 0.178 | 0.149 | -0.2 | 0.1 | -0.5 | 0.5 |  |  | C | | **0.012** | | 0.651 | 0.154 | 0.249 | 0.6 | | 0.1 | 0.4 | 0.3 |  |
|  | P | 0.538 | 0.971 | 0.090 | 0.892 | -0.2 | 0.0 | -0.6 | 0.0 |  |  | P | | 0.076 | | 0.638 | 0.175 | 0.248 | 0.5 | | 0.1 | 0.4 | 0.3 |  |
|  | LL | 0.824 | 0.795 | 0.314 | 0.427 | -0.1 | 0.1 | -0.4 | 0.3 |  |  | LL | | 0.191 | | 0.595 | 0.603 | 0.477 | 0.4 | | 0.2 | 0.2 | 0.2 |  |
|  | RL | 0.755 | 0.200 | 0.518 | 0.549 | 0.1 | -0.4 | -0.2 | 0.2 |  |  | RL | | 0.675 | | 0.177 | 0.844 | 0.750 | 0.1 | | -0.4 | 0.1 | -0.1 |  |
|  |  | ACC0 | RT0 | ACC2 | RT2 | ACC0 | RT0 | ACC2 | RT2 |  | |  | | ACC0 | | RT0 | ACC2 | RT2 | ACC0 | | RT0 | ACC2 | RT2 |  |
| **Beta_L_N0** | F | 0.223 | 0.223 | 0.490 | 0.889 | 0.4 | -0.4 | 0.2 | 0.1 | **Beta_L_N0** | | F | | 0.406 | | 0.448 | 0.588 | 0.571 | 0.2 | | -0.2 | 0.2 | -0.2 |  |
|  | C | 0.802 | 0.805 | 0.521 | 0.541 | 0.1 | -0.1 | -0.2 | -0.2 |  |  | C | | 0.672 | | 0.986 | 0.777 | 0.311 | 0.1 | | 0.0 | 0.1 | -0.3 |  |
|  | P | 0.874 | 0.652 | 0.372 | 0.145 | 0.1 | -0.2 | -0.3 | -0.5 |  |  | P | | 0.829 | | 0.887 | 0.642 | 0.792 | -0.1 | | 0.0 | -0.1 | -0.1 |  |
|  | LL | 0.433 | 0.836 | 0.515 | 0.642 | 0.3 | 0.1 | 0.2 | 0.2 |  |  | LL | | 0.771 | | 0.772 | 0.788 | 0.321 | 0.1 | | 0.1 | -0.1 | -0.3 |  |
|  | RL | 0.991 | 0.592 | 0.662 | 0.803 | 0.0 | -0.2 | -0.2 | 0.1 |  |  | RL | | 0.292 | | 0.125 | 0.809 | 0.332 | -0.3 | | -0.4 | -0.1 | -0.3 |  |
