# Supplementary material for: Alpha desynchronization during simple working memory unmasks pathological aging in cognitively healthy individuals
Source: PLoS One. 2019 Jan 2;14(1):e0208517. doi: 10.1371/journal.pone.0208517 (PMC6314588; doi:10.1371/journal.pone.0208517)
Supplement: S4 Table — (DOCX) [file pone.0208517.s004.docx]

| **S4 Table. Pearson's correlation between early (_E_) and late (_L_) power and behavioral (ACC and RT) during 2-back.** | | | | | | | | | | | | | | | | | | | | |
| --- | --- | --- | --- | --- | --- | --- | --- | --- | --- | --- | --- | --- | --- | --- | --- | --- | --- | --- | --- | --- |
|  |  | p | | | | r | | | |  |  | p | | | | r | | | |  |
| **CH-NAT** |  | ACC0 | RT0 | ACC2 | RT2 | ACC0 | RT0 | ACC2 | RT2 | **CH-PAT** |  | ACC0 | RT0 | ACC2 | RT2 | ACC0 | RT0 | ACC2 | RT2 |  |
| **Theta_E_N2** | F | 0.320 | 0.086 | 0.907 | 0.272 | 0.4 | -0.6 | 0.0 | -0.4 | **Theta_E_N2** | F | 0.439 | 0.916 | **0.028** | 0.836 | -0.3 | 0.0 | -0.7 | 0.1 |  |
|  | C | 0.459 | 0.078 | 0.819 | 0.259 | 0.3 | -0.6 | -0.1 | -0.4 |  | C | 0.239 | 0.507 | **0.002** | 0.228 | 0.4 | 0.2 | -0.8 | 0.4 |  |
|  | P | 0.431 | 0.051 | 0.624 | 0.343 | 0.3 | -0.6 | -0.2 | -0.3 |  | P | 0.394 | 0.916 | **0.038** | 0.539 | -0.3 | 0.0 | -0.7 | 0.2 |  |
|  | LL | 0.071 | 0.110 | 0.420 | 0.136 | 0.6 | -0.5 | 0.3 | -0.5 |  | LL | 0.341 | 0.633 | **0.030** | 0.669 | -0.3 | -0.2 | -0.7 | 0.2 |  |
|  | RL | 0.287 | **0.028** | 0.678 | 0.168 | 0.4 | -0.7 | -0.2 | -0.5 |  | RL | 0.285 | 0.763 | 0.254 | 0.941 | -0.4 | 0.1 | -0.4 | 0.0 |  |
|  |  | ACC0 | RT0 | ACC2 | RT2 | ACC0 | RT0 | ACC2 | RT2 |  |  | ACC0 | RT0 | ACC2 | RT2 | ACC0 | RT0 | ACC2 | RT2 |  |
| **Theta_L_N2** | F | 0.296 | **0.007** | 0.819 | 0.586 | 0.4 | -0.8 | 0.1 | -0.2 | **Theta_L_N2** | F | 0.581 | **0.049** | 0.604 | 0.821 | -0.2 | 0.6 | -0.2 | 0.1 |  |
|  | C | 0.484 | 0.235 | 0.797 | 0.581 | 0.3 | -0.4 | 0.1 | -0.2 |  | C | 0.562 | 0.096 | 0.442 | 0.330 | 0.2 | 0.6 | -0.3 | 0.3 |  |
|  | P | 0.249 | 0.050 | 0.936 | 0.233 | 0.4 | -0.6 | 0.0 | -0.4 |  | P | 0.422 | 0.075 | 0.583 | 0.489 | -0.3 | 0.6 | -0.2 | 0.2 |  |
|  | LL | 0.643 | 0.061 | 0.978 | 0.186 | 0.2 | -0.6 | 0.0 | -0.5 |  | LL | 0.330 | 0.102 | 0.642 | 0.856 | -0.3 | 0.5 | -0.2 | 0.1 |  |
|  | RL | 0.830 | **0.012** | 0.297 | 0.434 | 0.1 | -0.8 | -0.4 | -0.3 |  | RL | 0.178 | **0.022** | 0.705 | 0.775 | -0.5 | 0.7 | -0.1 | 0.1 |  |
|  |  |  |  |  |  |  |  |  |  |  |  |  |  |  |  |  |  |  |  |  |
| **CH-NAT** |  | ACC0 | RT0 | ACC2 | RT2 | ACC0 | RT0 | ACC2 | RT2 | **CH-PAT** |  | ACC0 | RT0 | ACC2 | RT2 | ACC0 | RT0 | ACC2 | RT2 |  |
| **Alpha_E_N2** | F | 0.837 | 0.195 | 0.131 | 0.708 | -0.1 | -0.4 | -0.5 | -0.1 | **Alpha_E_N2** | F | 0.468 | 0.306 | 0.619 | 0.671 | -0.3 | -0.4 | -0.2 | 0.2 |  |
|  | C | 0.674 | 0.418 | 0.095 | 0.890 | -0.2 | -0.3 | -0.6 | -0.1 |  | C | 0.883 | 0.410 | 0.939 | 0.447 | -0.1 | -0.3 | 0.0 | 0.3 |  |
|  | P | 0.891 | 0.361 | 0.147 | 0.689 | -0.1 | -0.3 | -0.5 | -0.1 |  | P | 0.996 | 0.864 | 0.959 | 0.202 | 0.0 | -0.1 | 0.0 | 0.4 |  |
|  | LL | 0.795 | 0.398 | 0.101 | 0.370 | -0.1 | -0.3 | -0.5 | -0.3 |  | LL | 0.935 | 0.167 | 0.614 | 0.754 | 0.0 | -0.5 | -0.2 | 0.1 |  |
|  | RL | 0.665 | 0.381 | 0.055 | 0.563 | -0.2 | -0.3 | -0.6 | -0.2 |  | RL | 0.994 | 0.417 | 0.988 | 0.507 | 0.0 | -0.3 | 0.0 | 0.2 |  |
|  |  | ACC0 | RT0 | ACC2 | RT2 | ACC0 | RT0 | ACC2 | RT2 |  |  | ACC0 | RT0 | ACC2 | RT2 | ACC0 | RT0 | ACC2 | RT2 |  |
| **Alpha_L_N2** | F | 0.545 | **0.035** | 0.819 | 0.469 | 0.2 | -0.7 | -0.1 | -0.3 | **Alpha_L_N2** | F | 0.088 | 0.107 | 0.833 | 0.645 | -0.6 | 0.5 | -0.1 | -0.2 |  |
|  | C | 0.646 | 0.114 | 0.766 | 0.670 | 0.2 | -0.5 | -0.1 | -0.2 |  | C | 0.333 | 0.120 | 0.536 | 0.685 | -0.3 | 0.5 | -0.2 | -0.1 |  |
|  | P | 0.695 | 0.119 | 0.405 | 0.464 | 0.1 | -0.5 | -0.3 | -0.3 |  | P | 0.319 | 0.092 | 0.411 | 0.975 | -0.4 | 0.6 | -0.3 | 0.0 |  |
|  | LL | 0.314 | 0.069 | 0.770 | 0.133 | 0.4 | -0.6 | -0.1 | -0.5 |  | LL | 0.363 | 0.066 | 0.614 | 0.931 | -0.3 | 0.6 | -0.2 | 0.0 |  |
|  | RL | 0.837 | 0.070 | 0.301 | 0.455 | 0.1 | -0.6 | -0.4 | -0.3 |  | RL | 0.224 | 0.124 | 0.578 | 0.908 | -0.4 | 0.5 | -0.2 | 0.0 |  |
|  |  |  |  |  |  |  |  |  |  |  |  |  |  |  |  |  |  |  |  |  |
| **CH-NAT** |  | ACC0 | RT0 | ACC2 | RT2 | ACC0 | RT0 | ACC2 | RT2 | **CH-PAT** |  | ACC0 | RT0 | ACC2 | RT2 | ACC0 | RT0 | ACC2 | RT2 |  |
| **Beta_E_N2** | F | 0.514 | 0.923 | 0.353 | 0.797 | -0.2 | 0.0 | -0.3 | 0.1 | **Beta_E_N2** | F | 0.917 | 0.190 | 0.305 | 0.691 | 0.0 | -0.5 | 0.4 | -0.1 |  |
|  | C | 0.380 | 0.855 | 0.328 | 0.661 | -0.3 | 0.1 | -0.3 | 0.2 |  | C | 0.364 | 0.597 | 0.369 | 0.951 | 0.3 | -0.2 | 0.3 | 0.0 |  |
|  | P | 0.255 | 1.000 | **0.049** | 0.992 | -0.4 | 0.0 | -0.6 | 0.0 |  | P | 0.687 | 0.811 | 0.097 | 0.762 | 0.1 | 0.1 | 0.6 | -0.1 |  |
|  | LL | 0.453 | 0.737 | 0.143 | 0.631 | -0.3 | -0.1 | -0.5 | -0.2 |  | LL | 0.866 | 0.370 | 0.341 | 0.410 | 0.1 | -0.3 | 0.3 | -0.3 |  |
|  | RL | 0.389 | 0.791 | 0.110 | 0.906 | -0.3 | -0.1 | -0.5 | 0.0 |  | RL | 0.963 | 0.374 | 0.051 | 0.451 | 0.0 | -0.3 | 0.6 | -0.3 |  |
|  |  | ACC0 | RT0 | ACC2 | RT2 | ACC0 | RT0 | ACC2 | RT2 |  |  | ACC0 | RT0 | ACC2 | RT2 | ACC0 | RT0 | ACC2 | RT2 |  |
| **Beta_L_N2** | F | 0.907 | 0.568 | 0.926 | 0.915 | 0.0 | -0.2 | 0.0 | 0.0 | **Beta_L_N2** | F | 0.398 | 0.977 | 0.233 | 0.094 | -0.3 | 0.0 | 0.4 | -0.6 |  |
|  | C | 0.971 | 0.745 | 0.963 | 0.792 | 0.0 | -0.1 | 0.0 | 0.1 |  | C | 0.490 | 0.762 | 0.347 | **0.041** | -0.2 | 0.1 | 0.3 | -0.7 |  |
|  | P | 0.365 | 0.268 | 0.731 | 0.777 | 0.3 | -0.4 | 0.1 | -0.1 |  | P | 0.155 | 0.082 | 0.488 | 0.164 | -0.5 | 0.6 | 0.2 | -0.5 |  |
|  | LL | 0.687 | 0.219 | 0.819 | 0.534 | 0.1 | -0.4 | -0.1 | -0.2 |  | LL | 0.700 | 0.335 | 0.079 | 0.168 | -0.1 | 0.3 | 0.6 | -0.5 |  |
|  | RL | 0.751 | 0.290 | 0.944 | 0.903 | 0.1 | -0.4 | 0.0 | 0.0 |  | RL | 0.076 | 0.401 | 0.135 | 0.123 | -0.6 | 0.3 | 0.5 | -0.5 |  |
